# Fluorescence of Esthetic Resin Composites: Spectrophotometry and Photography Analysis Techniques

**DOI:** 10.1055/s-0043-1772244

**Published:** 2023-10-10

**Authors:** Joana Cruz, Raquel Eira, Catarina Coito, Bernardo Sousa, Alexandre Cavalheiro

**Affiliations:** 1 Department of Operative Dentistry, Faculty of Dental Medicine, Universidade de Lisboa/Faculdade de Medicina Dentária da Universidade de Lisboa, Rua Professora Teresa Ambrósio, Cidade Universitária, 1600-277 Lisboa, Portugal

**Keywords:** fluorescence, spectrophotometry, photography analysis, esthetic resin composites

## Abstract

**Objectives** The aim of this study was to evaluate the fluorescence of nine esthetic resin composite materials using two methods: spectrophotometry and photography analysis.

**Materials and Methods** Three specimens were made for each shade of resin composite (61 shades from 9 resin composites), for a total of 183 specimens. To obtain a control group, the crowns of three sound human incisors were prepared to obtain both enamel and dentin specimens. Fluorescence was measured using two methods: (1) a Spectroshade Micro fluorescence spectrophotometer (MHT Optic Research, Niederhasli, Switzerland) and (2) a photograph analysis using Adobe Photoshop CC software (version 2019.0.0, Adobe Systems, Inc.).

**Statistical Analysis** The results were statistically analyzed with an analysis of variance ( *α* = 0.05) and with the Tukey–Kramer adjustment. The correlation between two techniques was analyzed by Pearson correlation test ( *α* = 0.05).

**Results** Fluorescence was highly influenced by the brand of the resin composite and less influenced by the shade (chroma), except for opaquer and incisal shades, and there was almost no difference in opacity, except for incisal shades. There was a weak ( *r* = −0.105) and statistically not significant correlation ( *p* *=* 0.145) between photography analysis and spectrophotometry techniques.

**Conclusion** The fluorescence of esthetic resin composites is more dependent on the brand than on the shade or opacity.

## Introduction

The color of a natural tooth is determined by a combination of the optical properties of dentin and enamel, 1 which results from the reflection and refraction of light on those tissues. 2 3

To mimic the chromatic harmony of the natural tooth, direct esthetic restorations have been made in layers with different resin composite masses with the same optical characteristics as natural enamel and dentin. 4 5 Hence, manufacturers offer resin composites in different shades with optical properties as similar as possible to those of natural teeth in terms of hue, chroma, value, opalescence, translucency, and fluorescence. 2 6 Nonetheless, selecting the appropriate resin composite shade or shades that simulate the optical characteristics of dentin and enamel remains one of the most challenging clinical tasks. 7

The organic components of natural teeth are responsible for the phenomenon of fluorescence, which occurs when a tooth absorbs shortwave ultraviolet (UV) light and reflects it back in the visible spectrum. Both enamel and dentin are fluorescent tissues, but dentin produces more intense fluorescence than enamel because it contains a greater amount of organic material. 8 For this reason, natural teeth display a predominantly white with light blue tonality (bluish white) under UV light and appear lighter and whiter in daylight (8% of the solar radiation spectrum is UV light). 6

In daily life, most people are exposed to many situations in which natural or artificial UV light is present. In these cases, the significant fluorescent contrast between the natural tooth structure and resin composite materials with lower fluorescence creates an undesirable and unesthetic effect. In these situations, the success of dental treatment could be determined by the fluorescence of the restorative material. 9 Moreover, the fluorescence of resin composites is reduced as the material ages, unlike human teeth, which do not lose any of their fluorescence. 10 11 Thus, the esthetic results of anterior tooth restorations may be significantly compromised by the choice of materials with low fluorescence. Thus, modern esthetic resin composites, which generally lack fluorescence, should incorporate fluorescent pigments like rare earth elements (i.e., ytterbium, cerium, terbium, europium, or thulium) to add fluorescence to the resin composite, equivalent to that of natural teeth. 12 13 Fluorescence not only noticeably increases the value or luminosity of restorations without compromising their translucency but also reduces the phenomenon of metamerism, which is evident in some restorations that often appear well integrated in one type of light but completely unintegrated in another. 14

The optical properties of resin composites have been evaluated and characterized in several studies in terms of hue, chroma, and opacity/translucency. 15 16 17 Such information, although often not very accurate, is also readily available from manufacturers. 18 However, some studies 19 20 have confirmed that fluorescence is an optical phenomenon that is independent of tooth color, and information is lacking about the fluorescence of the resin composites intended for use in highly esthetic restorations.

The purpose of this study was to evaluate the fluorescence of nine resin composite materials and to compare two methods of measuring fluorescence: the Spectroshade Micro fluorescence spectrophotometer (MHT Optic Research, Niederhasli, Switzerland) and photography analysis using Adobe Photoshop CC software (version 2019.0.0, Adobe Systems, Inc.). The null hypotheses of this study were as follows: 1) There are no significant differences among fluorescence measurement methods; 2) There are no significant differences in fluorescence among different brands of resin composites; 3) There are no significant differences in fluorescence among different shades of resin composites; and 4) There are no significant differences in fluorescence among different opacities of resin composites.

## Materials and Methods

Nine light-curing resin composites in a total of 61 shades were studied ( Table 1 ). A polytetrafluoroethylene mold was used to obtain specimens with a diameter of 10 mm and a thickness of 1.5 mm. The mold was placed over a glass slab, and the resin composite was applied and packed into the top of the mold. Another glass slab was placed over it and compressed. Three specimens were made for each shade of resin composite, for a total of 183 specimens. Polymerization was carried out with a light curing unit (Elipar S10, 3M ESPE Neuss, Germany) at a light intensity of 600 mW/cm ^2^ , activated for 40 seconds on the top and bottom surfaces. The constancy of the light intensity was measured with a radiometer (Model 100, Demetron Research Corporation, Kerr, California, United States). Additionally, to obtain a control group, the crowns of three sound human incisors were prepared with a trepan bur (Ø 8.0/6.9 mm) under air/water-spray refrigeration at 40.000 rpm to obtain both enamel and dentin specimens with similar dimensions to the resin composite specimens.

**Table 1:** Resin composites materials used

Brand name	Manufacturer	Opacity	Shade
Herculite XRV	KERR - Orange, California, United States	Enamel	A3
			A2
			A1
		Dentin	A1
			A2
			A3
			A3.5
Omnichroma	Tokuyama Dental, Tokyo, Japan	Universal	Universal
Enamel Plus HRI	Micerium Sp.A., Avegno, Italy	Enamel	A3
			A2
			A1
		Dentin	A3
			A2
			A1
		Opaque	IWS
		Incisal	OA
			OBN
Point4	KERR - Orange, California, United States	Enamel	A3
			A2
			A1
Simplishade	KERR - Orange, California, United States	Universal	LT
			Medium
Harmonize	KERR - Orange, California, United States	Enamel	A3
			A2
			A1
		Dentin	A3.5
			A3
			A2
			A1
		Incisal	Clear
			Ambar
			Grey
Spectra LV	Dentsply Sirona, New York, United States	Universal	A4
			A3.5
			A3
			A2
			A1
Filtek Supreme XT	3M ESPE, Dental Products Two Harbors, Minnesota, United States	Enamel	A3
			A2
			A1
			White
		Body	A3.5
			A3
			A2
			A1
			White body
		Dentin	A3
			A2
			A1
		Incisal	Ambar
			Blue
			Clear
			Gray
Filtek Universal	3M ESPE, Dental Products Two Harbors, Minnesota, United States	Universal	XW
			A3.5
			A3
			A2
			A1

Abbreviations: XW, extrawhite; OBN, Opalescent Blue Natural; OA, Opalescent Amber; PO, pink opaquer.

Fluorescence was measured using a Spectroshade Micro fluorescence spectrophotometer and a photograph analysis in Adobe Photoshop CC (version 2019.0.0).

### Fluorescence Spectrophotometer Study

For each group, one specimen at a time was placed and fixed in a black background under UV light (Discolux 18W, Osram Licht AG, Munich, Germany), and its fluorescence was measured with a spectrophotometer at three points (one central point, another one 1 mm from its periphery, and a third point in the middle of the line that joins the other two).

### Adobe Photoshop CC Program Study

For each group, one specimen at a time was placed in a box (with its inside walls painted black) under UV light. Subsequently, a Nikon digital camera D3400 with a 105 mm 1:2:8/ F5 Macro lens was used without a flash to capture a photograph of each specimen, placed in a predetermined position inside the box. Adobe Photoshop CC (version 2019.0.0) was used to analyze the central portion of each specimen, based on the following procedure: 1) Each digital image was opened in Adobe Photoshop CC; 2) In the central window, the option “Discard the embedded profile” was selected; 3) In the top bar, the “Filter” option was selected, followed by the “Camera Raw Filter” option, which caused a new window to appear; 4) In this new window on the top bar, the option “Color Classification Tool” was selected, and the cursor marked the points to be evaluated; 5) Three points were evaluated for each specimen: one central point, another 1 mm from its periphery, and a third in the middle of the line joining the other two; 6) The “b” value was taken from each of these locations, which corresponds to the degree of blue of the image at those points (the higher the “b” value of the image, the higher the specimen's fluorescence level).

### Statistical Analysis

The results were statistically analyzed using an analysis of variance (α = 0.05), and post hoc multiple comparisons with the Tukey–Kramer adjustment were performed to identify differences between different categories of each factor (resin composite, shade, and opacity). A multivariable model was run to find adjusted predictive effects and identify the interaction effects between the three factors. The correlation between photography analysis and spectrophotometry techniques was analyzed using Pearson correlation test (α = 0.05).

## Results

### Comparison between Resin Composites (Brands)

**Fig. 1:**
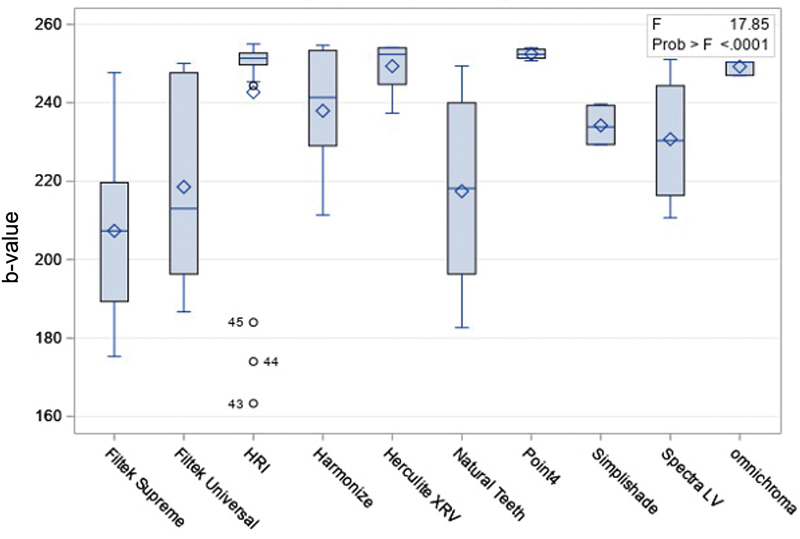
Fluorescence of resin composites brands and natural teeth, measured with the photographic analysis technique.

Using photography analysis technique, Filtek Universal (218.54 ± 24.21) was found to have significantly lower fluorescence than HRI (242.67 ± 25.11), Harmonize (237.92 ± 14.83), Herculite XRV (249.32 ± 6.08), and Point 4 (252.41 ± 1.32). Herculite XRV and Point4 had significantly higher fluorescence than natural teeth (217.44 ± 27.14). Spectra LV (230.71 ± 15.57) had significantly higher fluorescence than Filtek Supreme (207.35 ± 18.67) and Herculite XRV (249.32 ± 6.08). Filtek Supreme had significant lower fluorescence than Omnichroma (249.22 ± 6.08; Fig. 1 ).

**Fig. 2:**
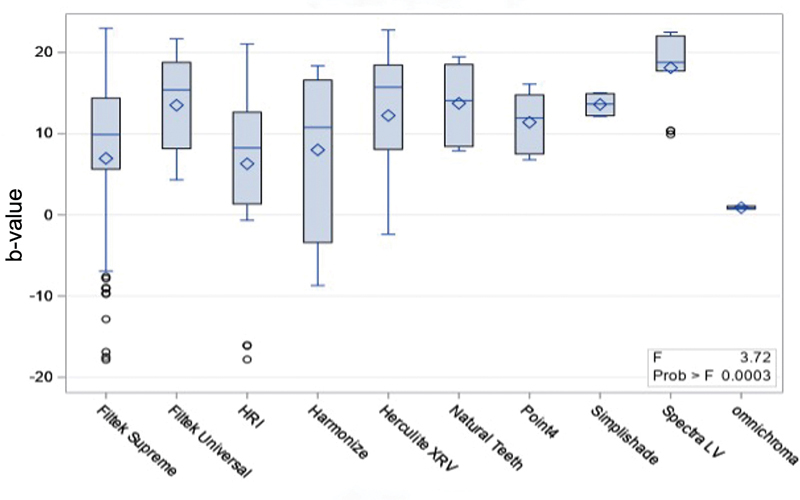
Fluorescence of resin composites brands and natural teeth, measured with the spectrophotometry analysis technique.

Based on spectrophotometry technique, Spectra LV (18.10 ± 4.45) had significantly higher fluorescence than Filtek Supreme (6.94 ± 11.28), HRI (6.29 ± 10.30), and Harmonize (8.00 ± 9.56; Fig. 2 ).

### Comparison between Shades

**Fig. 3:**
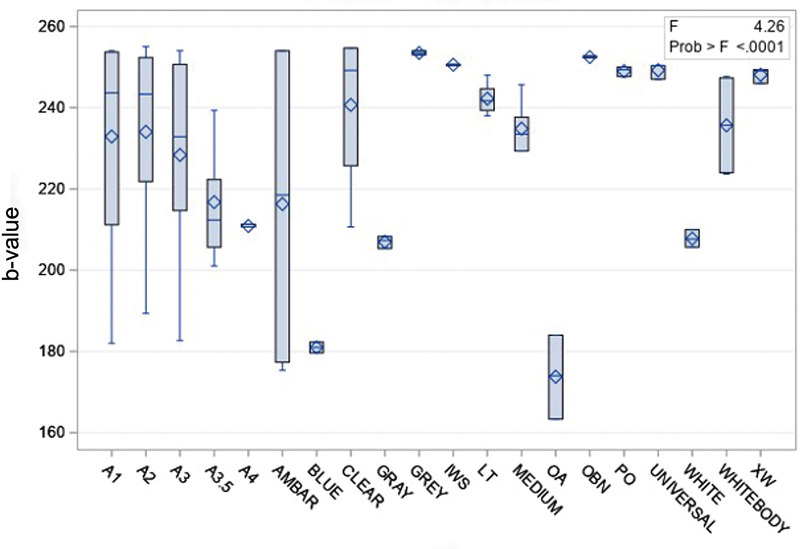
Fluorescence of different shades, measured with the photographic analysis technique.

Using photography analysis technique, OA (173.78 ± 10.34) had significant lower fluorescence than A1 (232.91 ± 23.93), A2 (234.05 ± 22.16), A3 (228.35 ± 23.95), Clear (240.67 ± 18.48), Gray (253.44 ± 0.51), IWS (250.56 ± 0.19), LT (242.22 ± 3.85), Medium (234.83 ± 6.62), PO (249 ± 1.20), OBN (252.44 ± 0.19), Universal (249.22 ± 1.92), White Body (235.67 ± 12.78), and XW (248.11 ± 1.84). Blue (181.00 ± 1.33) had significantly lower fluorescence than Clear (240.67 ± 18.48), Gray (253.44 ± 0.51), IWS (250.56 ± 0.19), LT (242.22 ± 3.85), Medium (234.83 ± 6.62), OBN (252.44 ± 0.19), PO (249 ± 1.20), Universal (249.22 ± 1.92), White body (235.67 ± 12.78), and XW (248.11 ± 1.84; Fig. 3 ).

**Fig. 4:**
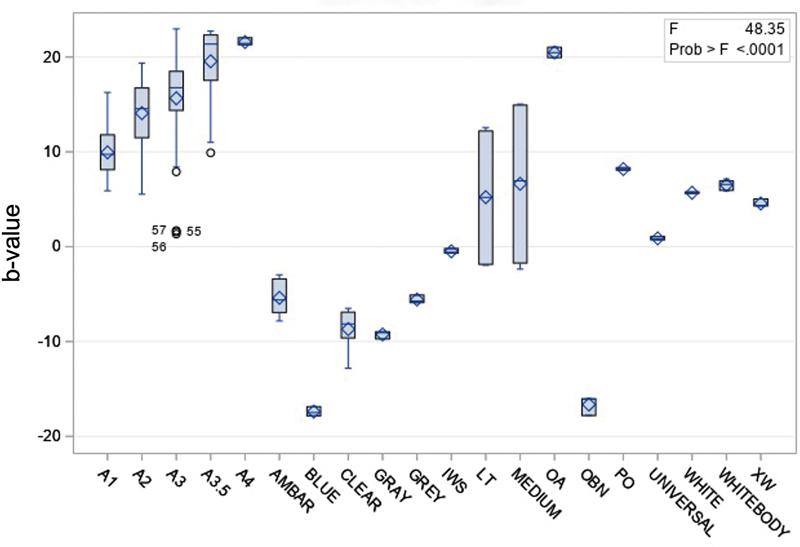
Fluorescence of different shades, measured with the spectrophotometry analysis technique.

Using spectrophotometry technique, many significant differences in fluorescence could be seen between different shades, particularly Ambar, Blue, Clear, Gray, IWS, AO, OBN, and PO, which were significantly different from most of the other shades ( Fig. 4 ).

### Comparison between Opacities

**Fig. 5:**
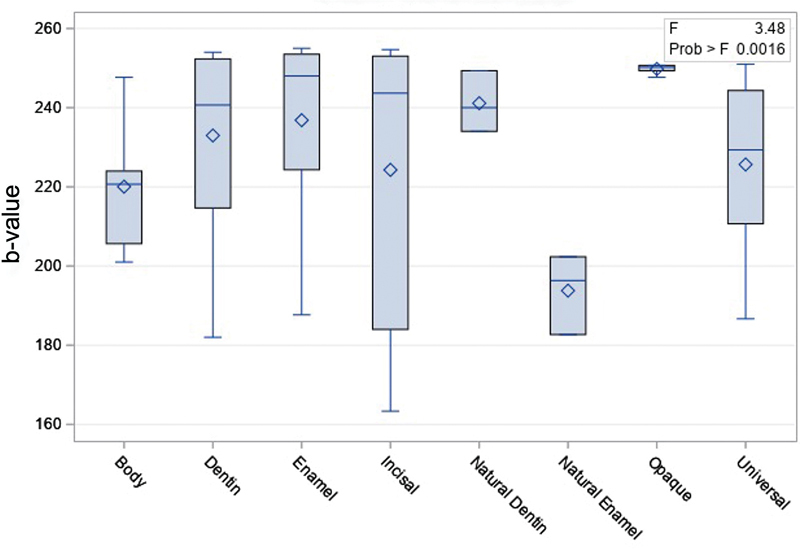
Fluorescence of different opacities, measured with the photographic analysis technique.

Using photography analysis technique, Opaque (249.78 ± 1.15) had significantly higher fluorescence than natural enamel (193.78 ± 10.08), and natural enamel had significantly lower fluorescence than enamel (236.83 ± 22.31; Fig. 5 ).

**Fig. 6:**
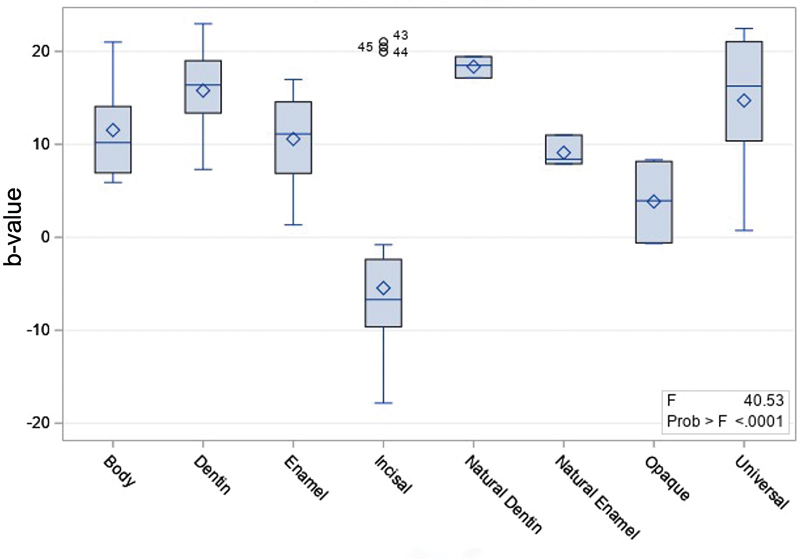
Fluorescence of different opacities, measured with the spectrophotometry analysis technique.

Using spectrophotometry technique, only incisal had significantly lower fluorescence (-5.46 ± 9.78; Fig. 6 ).

The results from the multivariable model analysis suggest that there are significant interaction effects among the type of resin composite and shade and opacity.

The Pearson correlation test revealed a weak ( *r* = −0.105) and statistically not significant correlation ( *p* *=* 0.145) between photography analysis and spectrophotometry techniques, especially for incisal shades like Ambar, Blue, or Clear and stains like IWS.

## Discussion

Fluorescence is an important optical property that influences the result of restorations and should not be overlooked when selecting a resin composite for clinical situations in which optimal esthetics are required. 4 10 21 To achieve optimal optical harmony between the tooth and final restoration, it is essential to use materials with fluorescence similar to that of natural teeth.

Most studies have used a spectrophotometer to perform fluorescence analysis of resin composites. 10 22 23 Recently, a study used a new methodology to analyze fluorescence: Adobe Photoshop CC. 9 This study utilized both techniques and found that the results obtained with the two techniques were not similar. These results are different from the study of Brokos et al, 24 who concluded that photography analyses technique resulted in photos with color coordinates that were highly correlated with the fluorescence intensities measured by a UV–visible spectrophotometer.

Ideal restorative materials should have fluorescence similar to that of natural teeth. 16 25 In the absence of fluorescence, the esthetic qualities of restorations could be compromised not only under UV illumination but also under daylight illumination. 26 In this study, the spectrophotometry technique revealed no differences between natural teeth and resin composites. Using photography analyses technique, Herculite XRV and Point4 exhibited more fluorescence than natural teeth.

It was also possible to verify that, regardless of the technique used, the evaluated systems from the same manufacturer generally present similar fluorescence levels with a characteristic fluorescence pattern, which may be explained by a specific concern and capability of certain manufacturers to include fluorescence components in their products. In this study, there were no differences in fluorescence between the most commonly used chroma (A1, A2, A3) in clinical practice, regardless of the resin composite brand.

The composition, size, and distribution of filled particles as well as the matrix phase and pigments employed determine the absorption, dispersion, and reflection of light by these materials. 27 Fluorescence will be given by the last layer of the material or, if this layer is very thin, by a combination of layers, resulting in intermediate levels of fluorescence. 4 8 For this reason, it is important that both enamel and dentin resin composites have fluorescence patterns similar to those of enamel and natural dentin. In this study, when evaluating opacity differences regardless of manufacturer and shade, it was found that resin composites do not exhibit substantially different values from natural enamel and dentin.

In this study, as in other similar studies, 10 16 17 no attempt was made to relate differences in fluorescence to compositional differences of the materials. Since no information is given by the manufacturers regarding what substances they are using to imitate natural tooth fluorescence, more studies are needed to relate the structure and composition of resin composites to their fluorescence properties. Nevertheless, fluorescence does not seem to be influenced much by the composite filler properties of specific composite brands, aside from the pigments, such as rare earth elements, which are specifically added to add fluorescence to the resin composite.

The extrapolation of the results of this type of *in vitro* study to a clinical situation is questionable. 28 Teeth and resin composite restorations are exposed to the full spectrum of sunlight radiation, which includes not only UV light but also visible light radiation or infrared radiation. The fact that fluorescence was evaluated solely by illuminating the specimens with a lamp emitting UV radiation, excluding all other types of radiation, is thus a limitation of the present study.

In the future, it would be interesting to verify whether esthetic resin composites with very thin layers have the same fluorescence characteristics.

## Conclusion

The results of this study require the rejection of the null hypotheses. The evaluations of fluorescence using the spectrophotometry and photography analysis techniques were not similar. Furthermore, there were differences between the different resin composites (brands). However, it was found that the most commonly used shades (A1, A2, and A3) exhibit similar fluorescence, and dentin and natural enamel have similar fluorescence to the dentin and enamel resin composites used in this study.
